# From Mr. Herbert J. Paterson, to the Editor of "The Times."

**Published:** 1919-06-14

**Authors:** Herbert J. Paterson

**Affiliations:** Medical Hon. Secretary, Royal British Nurses' Association, and Hon. Treasurer, Central Committee for State Registration of Trained Nurses.


### NURSES' REGISTRATION.

### The Central Committee's Supporters.

### FURTHER LETTERS.

THE following letters continue the correspondence in the *Times* which we published last week on page 251 :

### From Mr. Herbert J. Paterson, to the Editor of "The Times." (*See Ierne's article p.* 270.)

SIR,—In your issue of June 3 Sir Henry Burdett questions the figures with regard to the number of nurses represented by the Central Committee. I think it would be within the mark to state that the Central Committee represents by delegation about 35,000 medical men and nurses. At any rate, the number of trained nurses represented is not less than 13,000, and I have no doubt the various societies would be quite prepared to submit their evidence to the proper authorities.

I avoided purposely the use of the term "fully-trained," as at present there is no statutory definition of what constitutes a fully-trained nurse. If Sir Henry means three years' training in a general hospital, then certainly all our nurses do not come under this description, but neither do all of those on the College Register. In considering the numbers on the College Register, it is not unfair to call attention once more to the methods adopted to swell that register—methods which were the subject of adverse comment both in the House of Lords and in the House of Commons. I repeat once more that this is not a matter of numerical strength, but of justice. When the managers of the London training schools, most of whom in the past have consistently opposed State registration, support the Bill promoted by the College of Nursing, we may well say, "Timeo Danaos et dona ferentes." The primary aim of the Bill promoted by the College of Nursing appears to be—not State registration, but the incorporation of the college.

State registration is bound to come, in spite of the opposition of hospital governors, and it is not unfair to suggest, as the Marquess of Dufferin has done in the House of Lords, that "what was in their minds was that they should produce their own Bill with clauses which still kept the nurses under their domination." This is the vital point at issue. The college Bill is a hospital governors' and matrons' Bill—*i.e.*, an employers' Bill. The Central Committee's Bill is the Bill of the rank and file. Sir Henry Burdett states that this claim "won't bear examination," and the reason for this assertion appears to be that I am an employer of nurses. The line of argument is not obvious. It will be a surprise to medical men to learn that when, acting as agents, they engage a nurse they, *ipso facto*, become employers. It is hard to believe that any Court of Law would support such a contention. If, however, medical men are legally employers of nurses, then in the Central Committee's Bill the employers are represented on the General Nursing Council, and Sir Henry's argument therefore fails.

So far as I understand it, the last paragraph in Sir Henry's letter is an admission (1) that hospitals are employers of nurses; (2) that the nurses are sweated in order to provide nursing for the sick, which could not be provided otherwise. One of the results of a just Registration Bill will be to put an end to the present working conditions of nurses during their period of training.

As to the other points in my letter in which "there is no real substance," I am quite content to leave the decision to the judgment of those who read the full report of the debate in the House of Lords. In the opinion of many the able speeches of Lord Ampthill, Lord Dufferin and Lord Russell were very damaging to the College Bill. I am, yours faithfully,

HERBERT J. PATERSON, Medical Hon. Secretary, Royal British Nurses' Association, and Hon. Treasurer, Central Committee for State Registration of Trained Nurses.

(*The Times*, June 6.)

